# Optimal Management for Primary High Grade Ta Bladder Cancer: Role of re‐staging TURBT and Intravesical Adjuvant Therapy

**DOI:** 10.1002/bco2.363

**Published:** 2024-04-17

**Authors:** Tarek Ajami, Sunwoo Han, Ruben Blachman‐Braun, Helen Y. Hougen, Yuval Avda, Mark L. Gonzalgo, Bruno Nahar, Sanoj Punnen, Dipen J. Parekh, Isildinha M. Reis, Chad R. Ritch

**Affiliations:** ^1^ Desai Sethi Urology Institute University of Miami Miller School of Medicine Miami FL United States; ^2^ Biostatistics and Bioinformatics Shared Resource Sylvester Comprehensive Cancer Center University of Miami Miller School of Medicine Miami FL United States; ^3^ Department of Urology University of Iowa Hospitals and Clinics Iowa City IA United States; ^4^ Department of Public Health Sciences University of Miami Miller School of Medicine Miami FL United States

**Keywords:** non‐muscle invasive bladder cancer, progression‐free survival, recurrence‐free survival, restaging transurethral resection of bladder tumour

## Abstract

**Objective:**

This study aims to investigate the impact of risk group classification, restaging transurethral resection (re‐TURBT), and adjuvant treatment intensity on recurrence and progression risks in high‐grade Ta tumours in patients with non‐muscle invasive bladder cancer (NMIBC).

**Materials and methods:**

Data from a comprehensive bladder cancer database were utilized for this study. Patients with primary high‐grade Ta tumours were included. Risk groups were classified according to AUA/SUO criteria. Tumour characteristics and patient demographics were analysed using descriptive statistics. Cox proportional hazard regression models were used to assess the effect of re‐TURBT and other clinical/treatment‐related predictors on recurrence‐ and progression‐free survivals. The survivals by selected predictors were estimated using Kaplan–Meier method, and groups were compared by the log‐rank test.

**Results:**

Among 218 patients with high‐grade Ta bladder cancer, those who underwent re‐TURBT had significantly better 5‐year recurrence‐free survival (71.1% vs. 26.8%, *p* = 0.0009) and progression‐free survival (98.6% vs. 73%, *p* = 0.0018) compared with those with initial TURBT alone. Full BCG treatment (induction and maintenance) showed lower recurrence risk, especially in high‐risk patients. However, residual disease at re‐TURBT did not significantly affect recurrence risk.

**Conclusions:**

This study highlights the significance of risk group classification, the role of re‐TURBT, and the intensity of adjuvant treatment in the management of high‐grade Ta tumours. A risk‐adapted model is crucial to reduce the burden of unnecessary intravesical treatment and endoscopic procedures.

**Abbreviations**
BCG: Bacillus Calmette–Guerin
CIS: Carcinoma in situ
HG: High grade
HR: Hazards ratio
HR: High‐risk
IBCG: International Bladder cancer group
IR: Intermediate risk
NMIBC: Non‐muscle invasive bladder cancer
PFS: Progression‐free survival
RFS: Recurrence‐free survival
TURBT: transurethral resection of bladder tumour

## 1 INTRODUCTION

Non‐muscle invasive bladder cancer (NMIBC) accounts for 75% of bladder cancer cases. Standard management includes transurethral resection of bladder tumour (TURBT), which is both diagnostic and therapeutic.^1^, ^2^ Subsequent therapeutic procedures are based on a risk stratified approach, either performing a restaging‐TURBT (re‐TURBT) or initiating adjuvant intravesical therapy.

While more intensified treatment is recommended for high grade (HG) T1 tumours because of high risk of progression, management of HG Ta tumours remains controversial and with a lack of strong recommendations regarding re‐TURBT and intensity of adjuvant treatment.^3^ The main reasons for such variability are the non‐standardized risk classification of HG Ta disease as well as the heterogeneity of studies, which render the role of re‐TURBT and full adjuvant course as inconclusive. While this disease is considered as high risk in EAU guidelines before 2021 and International Bladder cancer group (IBCG) guidelines, both SUO/AUA^4^ and current EAU 2021 guidelines^1^ allocate Ta HG within the intermediate risk (IR) or high‐risk (HR) spectrum.

Herein, we investigate the influence of risk group, role of re‐TURBT, and the intensity of adjuvant treatment on the recurrence and progression risk of HG Ta tumours. We also seek to study the role of a synergistic effect of re‐TURBT on the efficacy of adjuvant treatment on recurrence‐ and progression‐free survivals.

## 2 PATIENTS AND METHODS

Data from our single institution NMIBC database (2010–2020) were retrospectively analysed. The study was approved by the institutional board review (IRB 2015110). For study purposes, we included only patients with primary HG Ta disease (AUA/SUO intermediate and high risk). A pathology review was done by genitourinary pathologists. We excluded patients with primary LG disease and those who did not complete follow up for at least one year with cystoscopy and or cytology. Tumour grading was based on WHO 2004/2016 classification.

Demographic and baseline information was recorded. Tumour characteristics (multiplicity, multifocality, and size) were obtained from cystoscopy or operative reports. Patients were counselled to undergo re‐TURBT within 6 weeks after the initial TURBT, especially if the initial TURBT was done in another institution or if muscle was not present in the initial specimen. The type of adjuvant treatment (either Bacillus Calmette–Guerin [BCG] treatment or intravesical chemotherapy) and intensity of BCG treatment (induction only or induction plus maintenance) were also collected from clinical records. An induction course was defined as at least five or six doses of intravesical therapy after transurethral resection of the bladder tumour. Maintenance was defined as a minimum of three doses given 3 to 6 months following induction. Re‐TURBT was performed at the discretion of the treating surgeon prior to initiating adjuvant intravesical treatment.

Patients were risk‐stratified based on the AUA/SUO risk classification^4^: intermediate risk for HG Ta tumours ≤3 cm and high risk for multifocal or >3 cm tumours or with concomitant carcinoma in situ (CIS). Patients who underwent re‐TURBT were classified if residual disease is present.

### 2.1 Outcome

Clinical outcomes were evaluated in terms of tumour recurrence or progression during follow‐up period. Recurrence was defined as the presence of any high‐grade tumour during follow‐up, and progression was defined as any stage progression (T1 or higher stages). Recurrence‐free survival (RFS) was defined as the elapsed time from date of the first TURBT for Ta HG bladder cancer to the first documented date of the presence of any positive tumour during follow‐up. Progression‐free survival (PFS) was defined as the elapsed time from the date of the first TURBT for Ta HG bladder cancer to the first documented date of any up‐staging tumour (T1 or higher stages) during follow‐up. Patients without events were censored at the last follow‐up visit.

### 2.2 Statistical Analysis

Analyses were performed in SAS 9.4 (SAS Inc., Cary, NC). Descriptive statistics were used to summarize demographic and baseline characteristics. Cox proportional hazard regression models were used to assess the effect of re‐TURBT and other clinical/treatment‐related predictors on RFS and PFS. Estimates of hazards ratios (HRs) with corresponding 95% confidence intervals and *p*‐values from the Wald test were reported. RFS and PFS by selected predictors were estimated using Kaplan–Meier method, and groups were compared by the log‐rank test. A *p*‐value <0.05 was considered statistically significant.

## 3 RESULTS

A total of 218 patients with HG Ta bladder cancer were identified as meeting inclusion criteria. The median age of diagnosis was 68.5 years (IQR: 59–76), 87 patients (40%) were classified as intermediate risk group, and 74 patients (33.9%) underwent re‐TURBT with residual disease detected in 42 (56.8%). Seven (16%) had persistent CIS, eight (19%) with LG Ta, and 20 (47%) with HG Ta; four patients (9.5%) were upstaged to T1 and none to T2. One hundred and seventy‐three patients (79.3%) received adjuvant treatment. Of those treated with BCG, 71 patients (43%) underwent full treatment schema (induction and maintenance of 1 year) while 91 (56%) received induction treatment only (Table 1).

**TABLE 1:** Characteristics of patients with Ta bladder cancer in the first TURBT.

Characteristic	Total
	N (%)
Total patients	218 (100)
Age (years)	
<70 years	116 (53.2%)
≥70 years	102 (46.8%)
Mean (SD)	67.2 (±11.8)
Median (p25, p75)	68.5 (59, 76)
Gender	
Male	163 (74.8%)
Female	55 (25.2%)
Risk group	
Intermediate	87 (39.9%)
High	131 (60.1%)
Number of tumours	
1	153 (70.2%)
≥2	65 (29.8%)
Tumour size	
<3 cm	139 (63.8%)
≥3 cm	79 (36.2%)
Tumour focality	
Unifocal	160 (73.4%)
Multifocal	58 (26.6%)
Re‐TURBT	
No	144 (66.15)
Yes	74 (33.9%)
MP at the 1st TURBT (N = 74)	
No	30 (40.5%)
Yes	44 (59.5%)
Stage at re‐TURBT (N = 74)	
Non‐T0 (residual disease, >T0)	42 (56.8%)
T0	32 (43.2%)
Post‐TURBT treatment	
None	45 (20.6%)
Chemotherapy	11 (5.0%)
BCG	162 (74.3%)
BCG modality (N = 162)	
Induction (6 weeks)	91 (56.2%)
Induction + Maintenance (1 year)	71 (43.8%)

BCG: Bacillus Calmette–Guerin; Re: restaging; TURBT: transurethral resection of bladder tumour; MP: muscle present.

SD: standard deviation; P25, p75: percentiles 25% and 75%.

**FIGURE 1:**
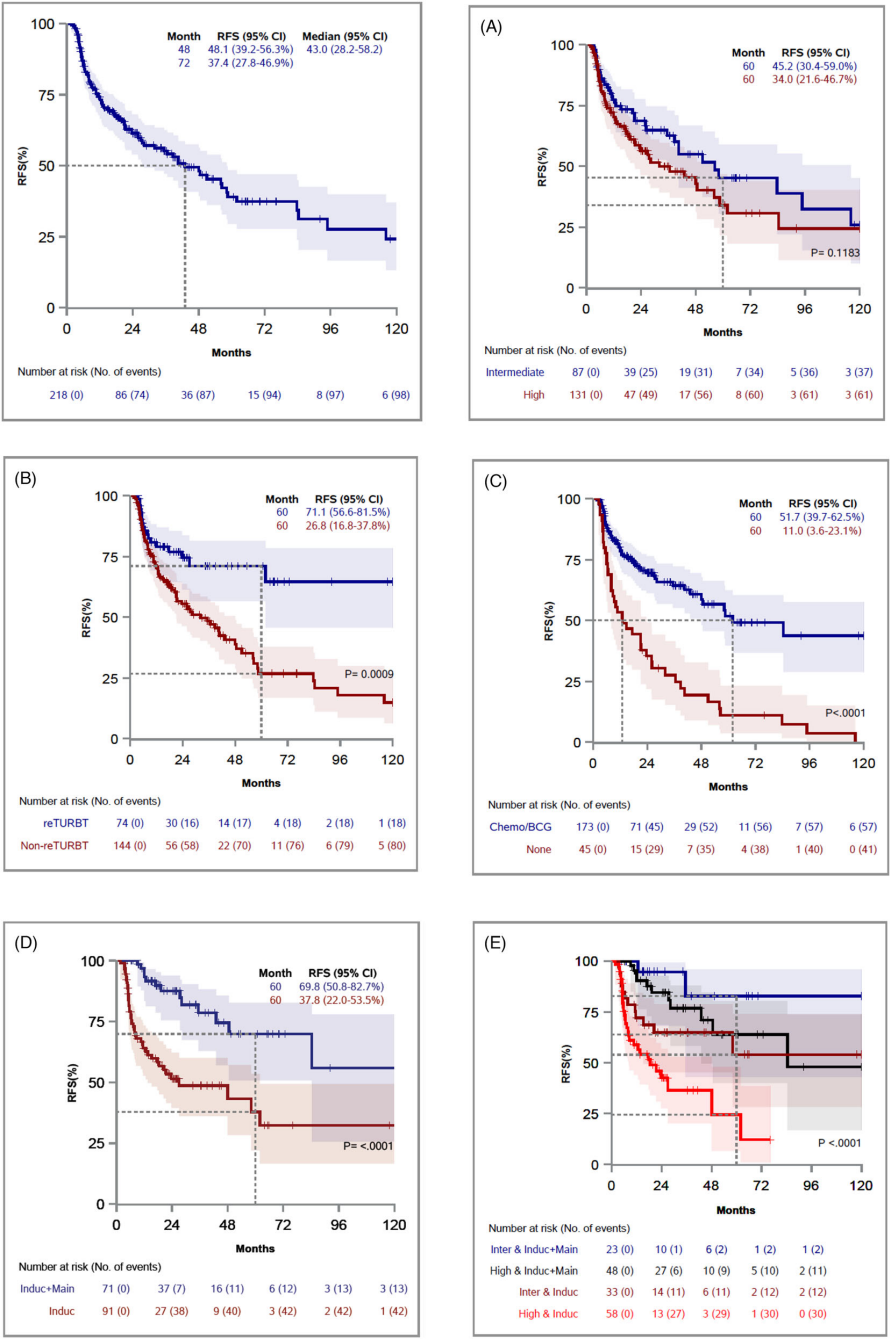
Kaplan–Meier curves for RFS, stratified by Risk group (A), Re‐TURBT (B), Adjuvant treatment (C), BCG modality (D; N = 162), and Risk group and BCG modality (E; N = 162).

**FIGURE 2:**
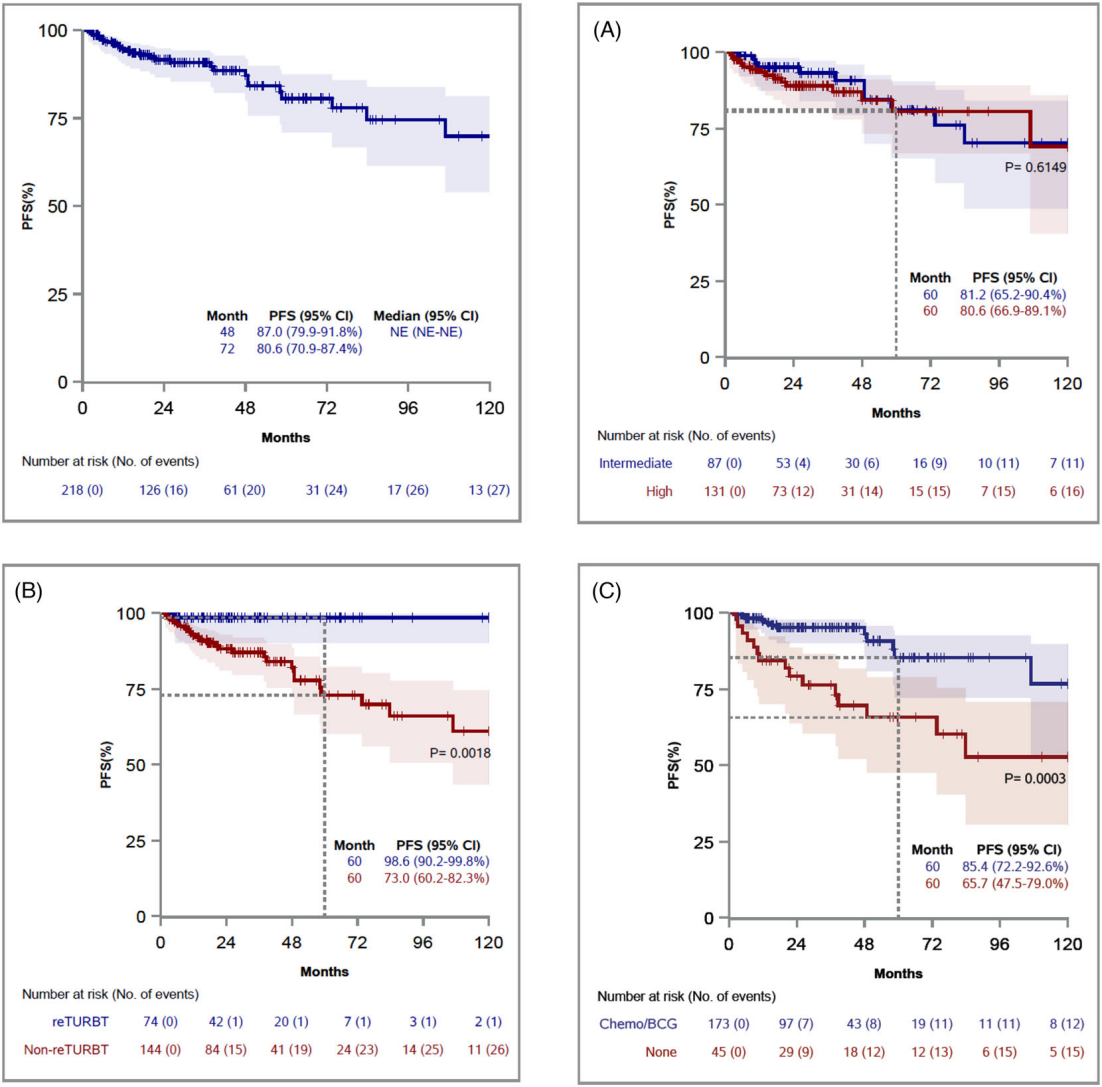
Kaplan–Meier curves for PFS, stratified by Risk group (A), Re‐TURBT (B), and Adjuvant treatment (C).

In overall cohort, 98 patients (45%) had recurrence (median RFS of 10.6 months [range = 1.7–116.2]), whereas 27 progressed (12.7%) (median PFS of 17.0 months [range = 1.7–106.9]). None of the patients presented extravesical progression. The remaining event‐free 120 patients (55%) were followed for median of 24.3 months (range = 3.1–120.0). Cox univariable analysis showed that risk group and tumour characteristics (number of tumours, size, and focality) were not significantly associated with RFS and PFS (*p* > 0.05). However, re‐TURBT and adjuvant treatment were significant predictors on RFS and PFS (*p* < 0.02) (Panel A in Tables 2 and 3). Patients who underwent re‐TURBT had longer RFS at 5 years (71.1% vs. 26.8%, *p* = 0.0009) and PFS (98.6% vs. 73%, *p* = 0.0018), compared with initial TURBT only (Figures 1B and 2B). On Cox multivariable analysis, re‐TURBT was a significant predictor of longer RFS and PFS, adjusting for risk group and adjuvant treatment. In particular, compared with intermediate risk patients, being high risk was independently associated with recurrence (HR = 1.77, 95% CI = 1.16–2.71, *p* = 0.008), but not with progression, most likely because of small number of progression events (HR = 1.90, 95% CI = 0.85–4.26, *p* = 0.118; based on 27 events) (Panel A in Tables 2 and 3).

**TABLE 2:** Univariable and multivariable Cox regression analyses assessing the effect of clinical/treatment‐related variables on recurrence‐free survival (RFS).

Models	Category	Univariable		Multivariable	
A. Overall cohort (98 events in N = 218)		HR (95%CI)	P	HR (95% CI)	*p*
Risk group	High vs. Intermediate (ref)	1.39 (0.92, 2.09)	0.120	1.77 (1.16, 2.71)	0.008
Number of tumours	≥2 vs. 1 (ref)	1.17 (0.75, 1.82)	0.483	‐‐	‐‐
Tumour size	≥3 cm vs. <3 cm (ref)	1.03 (0.68, 1.55)	0.907	‐‐	‐‐
Tumour focality	Multifocal vs. Unifocal (ref)	1.31 (0.84, 2.06)	0.232	‐‐	‐‐
Re‐TURBT	Yes vs. No (ref)	0.43 (0.26, 0.72)	0.001	0.58 (0.34, 1,00)	0.050
Adjuvant Treatment	BCG/Chemo vs. None (ref)	0.32 (0.21, 0.47)	<0.0001	0.33 (0.21, 0.51)	<0.0001

B. BCG group (55 events in N = 162 treated with BCG)		HR (95%CI)	*p*	HR (95%CI)	*p*
Risk group	High vs. Intermediate (ref)	1.87 (1.02, 3.44)	0.044	2.46 (1.32, 4.58)	0.005
Re‐TURBT	Yes vs. No (ref)	0.45 (0.24, 0.83)	0.010	0.45 (0.24, 0.82)	0.009
BCG modality	Induction + maintenance vs. Induction (ref)	0.27 (0.14, 0.50)	<0.0001	0.22 (0.12, 0.43)	<0.0001

BCG: Bacillus Calmette–Guerin; Re: restaging; TURBT: transurethral resection of bladder tumour; MP: muscle present. HR: Hazard ratio of recurrence for comparing groups. CI: confidence interval. *p*: *p*‐value testing HR = 1 from Wald test.

**TABLE 3:** Univariable and multivariable Cox regression analyses assessing the effect of clinical/treatment‐related variables on progression‐free survival (PFS).

Models	Unit	Univariable		Multivariable	
A. Overall group (27 events in N = 218)		HR (95%CI)	*p*	HR (95%CI)	*p*
Risk group	High vs. Inter (ref)	1.22 (0.56, 2.64)	0.616	1.90 (0.85, 4.26)	0.118
Number of tumours	≥2 vs. 1 (ref)	0.84 (0.33, 2.11)	0.704	‐‐	‐‐
Tumour size	≥3 cm vs. <3 cm (ref)	0.66 (0.28, 1.55)	0.337	‐‐	‐‐
Tumour focality	Multi vs. Uni (ref)	1.52 (0.65, 3.52)	0.335	‐‐	‐‐
Re‐TURBT	Yes vs. No (ref)	0.08 (0.01, 0.61)	0.015	0.11 (0.01, 0.80)	0.030
Adjuvant Treatment	BCG induc vs. None (ref)	0.38 (0.16, 0.90)	0.029	‐‐	‐‐
	BCG induc+main vs. None (ref)	0.10 (0.02, 0.44)	0.002	‐‐	‐‐
	BCG induc+main vs. induc (ref)	0.26 (0.06, 1.25)	0.093	‐‐	‐‐
	BCG/Chemo vs. None (ref)	0.27 (0.12, 0.57)	0.0007	0.32 (0.14, 0.70)	0.005

B. BCG group (10 events in N = 162 treated with BCG)		HR (95%CI)	*p*	HR (95%CI)	*p*
Risk group	High vs. Inter (ref)	2.43 (0.52, 11.46)	0.262	3.54 (0.71, 17.70)	0.124
Re‐TURBT	Yes vs. No (ref)	0.16 (0.02, 1.23)	0.077	0.15 (0.02, 1.17)	0.070
BCG modality	Induc+main vs. Induc (ref)	0.24 (0.05, 1.16)	0.076	0.18 (0.03, 0.98)	0.047

BCG: Bacillus Calmette–Guerin; Re: restaging; TURBT: transurethral resection of bladder tumour; MP: muscle present.

HR: Hazard ratio of progression for comparing groups. CI: confidence interval. *p*: *p*‐value testing HR = 1 from Wald test.

*Note:* Among 27 progression events, 19 with recurrence and progression simultaneously (within ±2 months) and eight with progression after recurrence (median elapsed time of 10.3 months [range: 6.7–58.3]). seventy‐one recurrence events without progression were censored in this analysis.

In the subset of 162 patients who underwent BCG treatment (Panel B in Tables 2 and 3), Cox univariable analysis found that BCG induction plus maintenance (vs. BCG induction only) was significantly associated with lower risk of recurrence (HR = 0.27, 95% CI = 0.14–0.50, *p* < 0.001), but not significant for PFS (HR = 0.24, 95% CI = 0.05–1.16, *p* = 0.076). However, in the multivariable model adjusting for risk group and re‐TURBT, maintenance therapy was significantly associated with a lower likelihood of recurrence (HR = 0.22, 95% CI = 0.12–0.43, *p* < 0.0001) and progression (HR = 0.18, 95% CI = 0.03–0.98, *p* = 0.047). Based on risk group and BCG modality, four groups were compared using the log‐rank test. Overall comparison was significant (*p* < 0.0001). Among selected four pairwise comparisons with Bonferroni correction, RFS was significantly better for BCG induction plus maintenance than BCG induction alone in the high‐risk group (adjusted *p* < 0.0001, Figure 1F black vs. red curves). However, there was no significant difference in RFS by BCG intensity in intermediate‐risk patients (adjusted *p* = 0.431, Figure 1F blue vs. dark red curves). As expected, RFS was significantly better for intermediate risk group than high risk group in patients who received BCG induction only (adjusted *p* = 0.004, Figure 1F dark red vs. red curves).

In the subset of 74 patients who underwent re‐TURBT, there were 18 recurrence events (Panel C in Table 2). The absence of residual disease at re‐TURBT was not associated with risk of recurrence in univariable (HR = 1.21, 95% CI = 0.47–3.14, *p* = 0.693) and multivariable (HR = 1.37, 95% CI = 0.52–3.59, *p* = 0.525) analyses.

## 4 DISCUSSION

The optimal management of HG Ta tumours represents an unmet need in clinical practice because of the heterogeneity of the disease within NMIBC, challenges with BCG shortage and burden of repeated endoscopic resections. In this study, we aimed to decipher the role of restaging TURBT as well as intensity of BCG adjuvant therapy on the risk of recurrence and progression in primary HG Ta tumours. In concordance with AUA/SUO guidelines, we report a significant benefit for both restaging TURBT and full induction plus maintenance in high risk NMIBC.

High grade, intermediate risk NMIBC occurs less commonly than high‐grade, high‐risk disease and there is some controversy as to whether all HG tumours should be included in the same risk group. While both entities are HG, some intermediate risk patients may be over treated with full intensity BCG and exposed to added morbidity of frequent surveillance regimens. Bree et al.^5^ advocates for considering all HG Ta as high risk given similar survival outcomes between EAU 2021 intermediate and high risk classified HG Ta tumours adequately treated with BCG. Furthermore, no differences were found when considering the number of risk factors to distinguish between IR and HR according to the same guidelines. We report similar findings in this aspect, as individual tumour characteristics do not affect the prognosis. However, when considering risk stratification according to AUA/SUO classification, we found that HG IR tumours had lower recurrence rates after adjusting with adjuvant treatment compared with HR.

Recommendations regarding repeat TURBT vary between guidelines: the AUA/SUO guideline recommends re‐TURBT in all high‐risk tumours, whereas the EAU guideline endorses performing re‐TURB based on the presence of muscularis propria in the initial TURB. The beneficial role of re‐TURBT in HG Ta has been addressed in previous publications^6^, ^7^. In two series, restaging TURB is prognostic and improved recurrence and progression free survivals. However, these analyses did not incorporate adjuvant BCG intensity when considering efficacy of re‐TURBT. To our knowledge, this is the first study to analyse the role of re‐TURB among different BCG treatment schemas (induction alone vs. induction plus maintenance). Hensley et al.^7^ included patients with adequate BCG (full induction plus at least one maintenance) while Sfakianos et al.^6^ studied the role of re‐TURB in patients with high grade NMIBC only treated with induction course but no stratification was done based on stage. In line with the findings reported by Hensley et al.,^7^ our study did not find the presence of residual disease on re‐TURBT to be prognostic. Nevertheless, other studies have shown that certain adverse features on re‐TURBT (high grade, lymphovascular invasion, multifocality) are predictors for BCG response^8^.

The potential role of deintensification of adjuvant treatment in HG Ta NMIBC remains controversial.^9^ Several trials and meta‐analyses have attempted to address the optimal duration of BCG treatment and have shown that shorter duration of maintenance may be sufficient for intermediate risk disease.^10^ Results of EORTC‐GU Cancers group Study of maintenance BCG showed no benefit of 3‐year maintenance over 1 year in case of EORTC intermediate risk group.^11^ Lamm et al.^12^ showed benefit of maintenance BCG compared with standard induction treatment but without stratification based on stage or grade. Notably, the intermediate risk group in these studies did not specifically include HG Ta disease. Nevertheless, the results of these studies have been extrapolated to AUA/SUO guideline recommendations regarding management of intermediate risk disease and a shorter course of maintenance. While our study did not specifically address the duration of maintenance, we found that in HG intermediate risk disease, induction only may be sufficient treatment as opposed to high‐risk disease where more intense adjuvant BCG (induction plus maintenance) is associated with better outcomes.^12^

There are several limitations to our study, specifically the retrospective nature and selection bias affecting treatment regimens (re‐TURBT and use of BCG). While we were able to classify use of maintenance, we did not have granular detail on the number of maintenance courses or adherence to specific protocols (e.g., SWOG regimen^12^). The decision for full treatment scheme and re‐TURBT was based on physician preference and was not homogeneous among providers. Another limiting factor is the non‐standardized detection of CIS using randomized biopsy and subsequently the risk of group misclassification. Finally, the low number of events in terms of progression could limit the analysis of the role of adjuvant therapies in this aspect.

## 5 CONCLUSION

The current study supports the risk stratification of HG‐Ta tumours based on AUA/SUO criteria for predicting recurrence and progression. In addition, our findings underscore the importance of a risk‐based approach when performing re‐TURBT and determining the intensity of adjuvant BCG therapy in the management of these tumours. Risk stratification is a critical aspect in the management of NMIBC and is essential to reduce the potential morbidity and burden of over‐treatment and endoscopic procedures.

## AUTHOR CONTRIBUTIONS

Tarek Ajami had full access to all the data in the study and takes responsibility for the integrity of the data and the accuracy of the data analysis.

Study concept and design: Ajami, Hougen, Ritch.

Acquisition of data: Ajami, Ritch, Han, Hougen.

Analysis and interpretation of data: All authors.

Drafting of the manuscript: All authors.

Critical revision of the manuscript for important intellectual content: all authors.

Statistical analysis: Han, Reis.

Obtaining funding: None.

Administrative, technical, or material support: None.

Supervision: Ritch.

Other: None.

## CONFLICT OF INTEREST STATEMENT

The authors declare no conflicts of interest.
